# A novel CuBi_2_O_4_/polyaniline composite as an efficient photocatalyst for ammonia degradation

**DOI:** 10.1016/j.heliyon.2022.e10210

**Published:** 2022-08-18

**Authors:** Nafees Ahmad, Jerry Anae, Mohammad Zain Khan, Suhail Sabir, Pablo Campo, Frederic Coulon

**Affiliations:** a School of Water, Energy and Environment, Cranfield University, Cranfield, MK 43 0AL, UK; b Environmental Research Laboratory, Department of Chemistry, Aligarh Muslim University, Aligarh, India, 202002; c Department of Chemistry, Integral University, Lucknow, India, 226026

**Keywords:** Photocatalyst, In-situ polymerization, Kinetics, Scavengers, Electron-hole pairs

## Abstract

A novel polyaniline (PANI) coupled CuBi_2_O_4_ photocatalyst was successfully synthesized via in situ polymerization of aniline with pre-synthesized CuBi_2_O_4_ composites. The structure and morphology of the synthesized CuBi_2_O_4_/PANI composite photocatalyst were characterized by X-ray diffraction (XRD) and Fourier transform infrared spectroscopy (FTIR) and the photocatalytic performance were evaluated through degradation process of ammonia in water under visible light irradiation. The resultant CuBi_2_O_4_/PANI composite showed exceptional stability as its structure and morphology persisted even after being immersed in water for 2 days. The composite photocatalyst exhibited improved charge transport properties due to the electrical conductivity of the PANI protective layer, leading to enhanced photoelectrochemical activity in water and removal of ammonia. PANI with CuBi_2_O_4_ (10% wt) heterostructure was applied for photodegradation of ammonia and exhibited a 96% ammonia removal efficiency (30 mg/l with 0.1 g photocatalyst and 180 min), as compared to PANI (78%) and CuBi_2_O_4_ (70%). The degradation was attributed to the efficient charge transfer (e^−^ and h^+^) and formation of reactive oxygen species upon simulated sunlight exposure. The present work suggests that the CuBi_2_O_4_/PANI photocatalyst can be synthesized in a simple process and provides an excellent adsorption capacity, high photocatalytic activity, long term stability, and reusability making it a promising alternative for ammonia removal from wastewater.

## Highlights

• Novel CuBi_2_O_4_/PANI composite was synthesized via *in situ* polymerization of aniline.
• High surface area of CuBi_2_O_4_/PANI (45 m^2^g^-1^) exhibits higher efficiency towards adsorption and photocatalytic degradation of ammonia.
• Material shows synergistic effect by improving the redox ability for enhanced photodegradation of ammonia.
• Holes (h^+^) and hydroxyl radicals (·OH) were confirmed as the prime reactive species for the photodegradation of NH_3_.

## Graphical abstract

**Figure:**
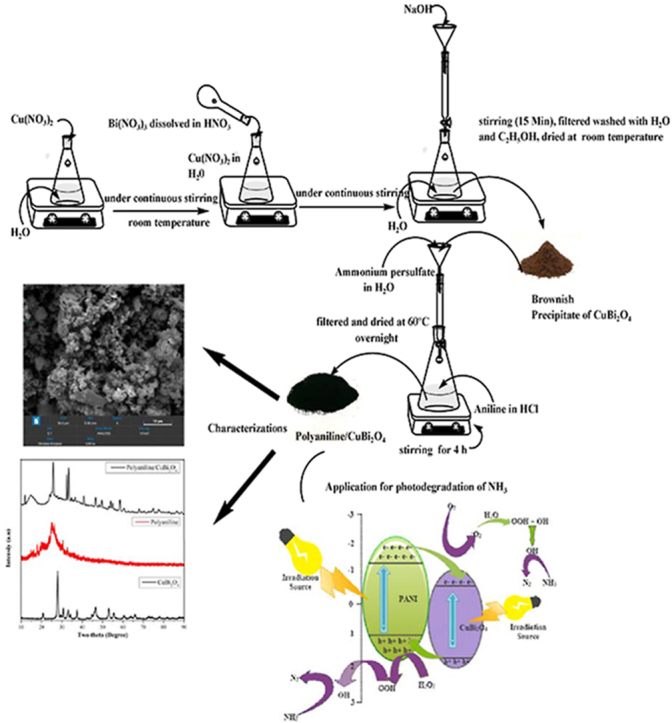

## 1 Introduction

Ammonia (NH_3_ or NH_4_^+^) can have significant effect on both the environment and human health and negatively affect biodiversity. The main release source of ammonia in the environment is from agriculture activities such as man-made fertilizer application, fossil fuel, manure, and slurry management (Tang et al., 2021; Bao et al., 2022; Chai et al. 2021a, 2021b). High concentration of ammonia in water has detrimental effects leading to eutrophication or changes in species and reduces the efficiency of chlorine disinfectants in drinking water (Liu et al., 2017; Zhang et al., 2020).

Several treatment methods including air stripping (Ukwuani and Tao, 2016), ion exchange (Almutairi and Weatherley, 2015), adsorption (Qiang et al., 2020), biological nitrification-denitrification (Li et al., 2019) and breakpoint chlorination (Pressley et al., 1972) have been widely adopted for ammonia removal. These technologies, however, show drawbacks such as air stripping, which requires large amount of photocatalysts along with high energy consumption and further generate secondary air emissions to be treated (Ukwuani and Tao, 2016). Biological nitrification-denitrification is a relatively slow process that is susceptible to changes in temperature, dissolved oxygen, and pH (Ye et al., 2019). In physical process such as ion exchange, physical adsorption and air stripping, ammonia can only be transformed from the aqueous (NH_4_^+^-N) to vapour phase (NO_3_^-^-N) rather than being converted to N_2_ (Qiang et al., 2020). Hence the need of alternatives for removing NH_4_^+^-N from wastewater at lower energy and chemical costs.

Photocatalysis has been shown as a promising green technology that can degrade organic pollutants in water and reduce or oxidize inorganic pollutants (Ren et al., 2021). However, catalysts are often prone to self-etching during use. Studies have therefore focused on the construction of heterojunction structures and doping to improve performance and to restore photocatalytic performance by oxidative reduction of deactivated photocatalysts. In this regard, a few semiconductor nanoparticles and nanocomposites such as g-C_3_N_4,_ SiO_2_-BiOCl and reduced graphene oxide encapsulated into polymer matrices such Polyaniline (PANI), polypyrrole, polythiophene and polyfuran have been used (Sethuraman et al., 2021; Tian et al., 2017; Zheng et al., 2021; Yan et al., 2022; Ahmad et al. 2017, 2019**)**. Among the polymer matrices, PANI has shown excellent photocatalytic performance because of its redox properties, tuneable bandgap energy with suitable configuration of conduction band (CB)-valence band (VB), higher surface area, and better cost-effectiveness than nanoparticles (Xiong et al., 2022; Tanwar et al., 2017; Xu et al., 2017). Conversely, recombination of photo-induced electron-hole pairs can only happen when PANI is irradiated during photocatalysis, which limits the efficacy of the process and removal of organic contaminants (Kundu et al., 2017; Belabed et al., 2021). To overcome the issue of recombination of photo-induced electron-hole, heterojunction between PANI and the semiconductor is often recommended.

Until now, the use and application of polymer nanocomposites for the photocatalytic degradation of ammonia is limited. In this study, a novel CuBi_2_O_4_/PANI composite with photocatalytic activity is synthesized for the degradation of ammonia under the influence of light emitted diode (LED) irradiation. A series of photocatalysts were synthesized by in-situ polymerization of aniline and CuBi_2_O_4_ with a low temperature method to assess their performance for the removal of ammonia in water. A 15 W LED has been used as the irradiation source which consume less power supply than other UV and visible light sources. The factors affecting the adsorption capacity and photodegradation efficiency such as ammonia load, catalyst concentration, and the radiation time, as well as the PANI and CuBi_2_O_4_ content were investigated for process optimisation and mitigation strategies.

## 2 Materials and method

### 2.1 Chemicals and reagents

All the chemicals used in the study were of analytical grade. Ammonium persulfate (NH₄)₂S₂O₈), aniline C6H5NH2, hydrochloric Acid (HCl) (37%), copper nitrate tri hydrate (CuNO3.3H2O), bismuth nitrate penta hydrate (BiNO_3_.5H_2_O), sodium hydroxide (NaOH), nitric Acid (HNO_3_) (98%), ammonia (NH_3_), Nessler's Reagent (K_2_HgI_4_) (alkaline solution of mercuric (II) iodide and potassium iodide) were procured from Sigma Aldrich (USA).

## 3 Experimental section

### 3.1 Synthesis of the CuBi_2_O_4_

Synthesis of CuBi_2_O_4_ was carried out in accordance to Wang et al. (2015). Briefly, a solution of Cu(NO_3_)_2_•3H_2_O (1.5 mmol) in water was mixed with 3 mmol of Bi(NO_3_)_3_•5H_2_O, which was prepared beforehand in a nitric acid solution (0.1 M), under continuous stirring for 15 min at room temperature. Then10 ml of NaOH (6 M) was added to the solution and stirred for 30 min at room temperature. The crystals were then separated by filtration and sequentially washed three times with 10 ml of deionised water followed by ethanol. The filtrate was oven dried overnight at 60 °C. The synthesis was done twice before performing the characterisation analyses and the photocatalytic experiments.

### 3.2 Synthesis of PANI and CuBi_2_O_4_/PANI via oxidative polymerization method

**Figure 1:**
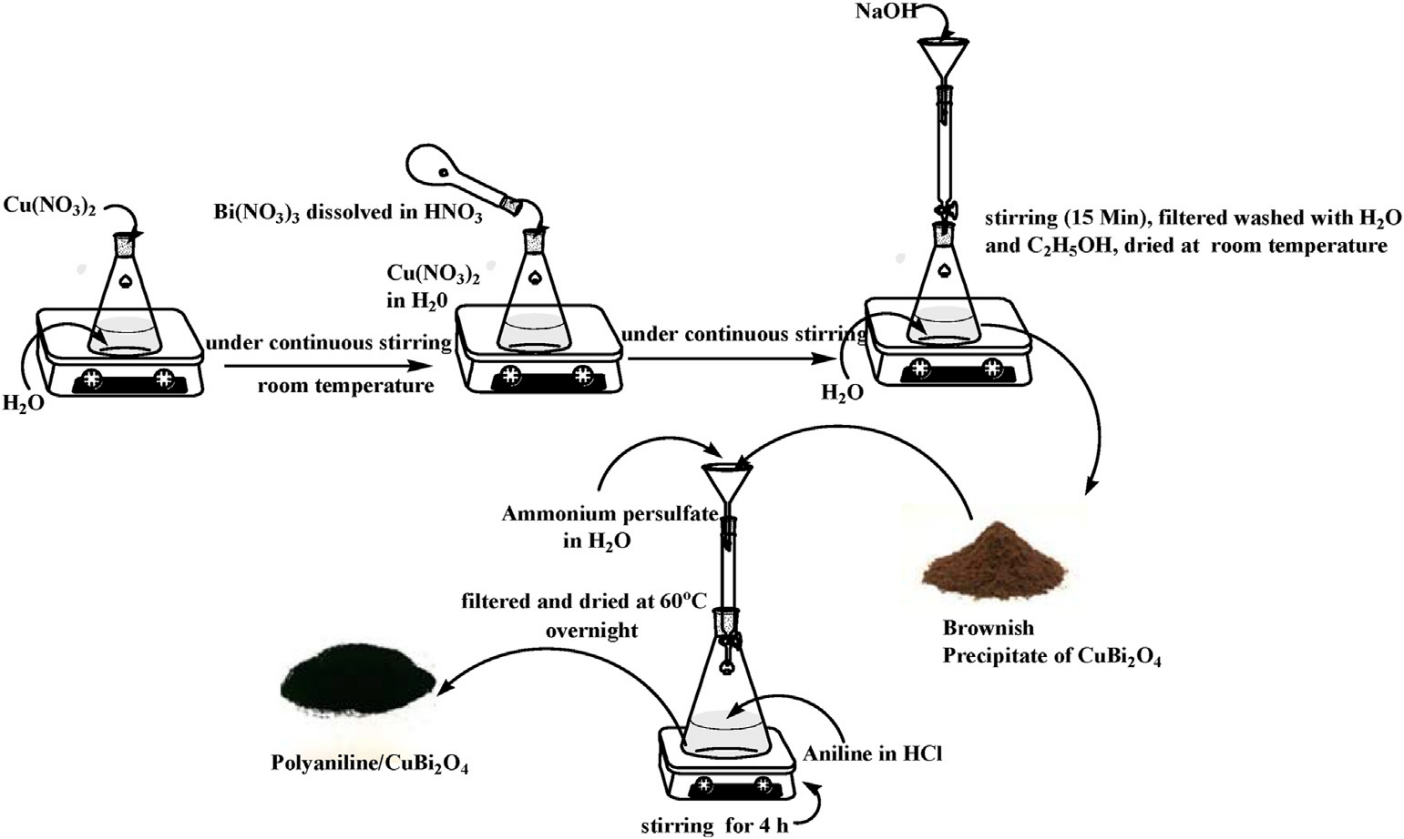
Overview of the steps for the synthesis of CuBi_2_O_4_/PANI composite.

PANI was synthesized by chemical oxidative polymerization of the aniline monomer with ammonium persulphate (Ahmad et al., 2019a, Ahmad et al., 2019b). A 1:1 solution of aniline (25 ml, 0.1 M prepared in 1 M HCl) and ammonium persulfate (25 ml, 0.1 M prepared in distilled water) were used for synthesis. Ammonium persulfate was added dropwise to the aniline solution and stirred for 4 h. A dark green precipitate was obtained and separated with a 150 mm filter paper (Whatman pore size 11 μm). The precipitate was then washed with 10 ml of distilled water followed by 10 mL of acetone and dried overnight at 60 °C. The PANI/CuBi_2_O_4_ composite was prepared with the same method in which pre-synthesized CuBi_2_O_4_ (1:3 (monomer to CuBi_2_O_4_) was added and sonicated for 15 min. The ratio of CuBi_2_O_4_ to aniline monomer was 1:3. An overview of the steps for the synthesis of PANI/CuBi_2_O_4_ composite is shown in Figure 1.

### 3.3 Composite materials characterisation

The crystal structure and purity of PANI, CuBi_2_O_4_ and CuBi_2_O_4_/PANI were established with a Siemens D5005 X-ray diffractometer (XRD). Surface morphology and elemental composition of the materials were obtained by scanning electron microscopy (SEM) coupled with energy dispersive X-ray spectroscopy (Tescan VEGA 3); size and shape of the particles were determined by transmission electron microscopy (TEM) (JEM 1400, JEOL, Japan). A PerkinElmer ultraviolet-diffuse reflectance Win Lab spectrometer (UV-DRS) was used to obtain the bandgap energy of the photocatalysts. The recombination of charge carriers during the photodegradation was evaluated from the fluorescence spectra (Horiba Scientific, Spectrofluorometer). The elemental states of the photocatalysts were determined by X-ray photon spectroscopy (XPS) (Kratos Axis Supra- X ray source (Mono Al kα) and energy -1486.7 eV) and the surface area, pore size and pore volumes were calculated by Brunauer–Emmett–Teller (Quantachrome NOVA 4000e). Ammonia concentrations were measured with an UV–VIS spectrophotometer (Jenway 6715, UK) with the Nessler's reagent as reported by Liu et al. (2018).

### 3.4 Photocatalytic activity and recyclability

The photocatalytic activity was evaluated by examining the photodegradation of ammonia in a 100 ml photoreactor equipped with visible light irradiation lamp (15 W LED). A mixture of 0.1 g of photocatalyst and 100 ml of the ammonia solution in the reactor was stirred continuously for 3 h with a magnetic bar till the end of the experiment. An air pump (Victsing aquarium air pump) with constant air flow was also used to supply atmospheric oxygen. Prior to LED irradiation, the solution was stirred for 20 min in the dark to attain equilibrium between the ammonia and the photocatalyst. Thereafter, the whole mixture was irradiated, and 5-ml aliquots were collected every 30 min to measure ammonia's concentration. Ammonia concentration was determined using the Nessler's reagent. Photodegraded sample of ammonia was determined by UV-visible spectrophotometer at maximum wavelength of 382 nm. A blank experiment was also carried out in which no photocatalyst was added. All conditions were carried out in triplicate. The absorbance was recorded between 300-600 nm. The degradation efficiency was calculated with Eq. (1):

(1) $$\text{Degradation ​efficiency ​}\left(\text{\%}\right)\text{ ​}=\text{ ​}\frac{C_{0}-C_{t}}{C_{0}}\times 100$$

where *C*_*0*_ is the initial concentration of the ammonia at equilibrium and *C*_*t*_ is the concentration at a given time *t*.

The stability and durability of the photocatalyst PANI coupled with CuBi_2_O_4_ were also investigated. Hence after each ammonia degradation experiment, the photocatalyst was recovered, washed with distilled water and acetone to remove any impurities, and dried overnight at room temperature. The photocatalyst was used in five consecutive cycles to check the durability.

To study the effect of the pH on the photodegradation of ammonia, the photocatalytic experiments were carried out at pH 5, 7, 9, 11 and 13. The pH was adjusted with 0.1 M solutions of either NaOH or HCl before the addition of the photocatalysts; each pH condition was tested in duplicate.

### 3.5 Degradation mechanisms

To identify the main reactive species responsible for the photodegradation of ammonia, *t*-butyl alcohol (TBA) and disodium ethylenediaminetetraacetic acid (EDTA) scavengers were used as quenching agents for •OH and h^+^, respectively. Both TBA (0.5 ml) and EDTA (0.025 g) were added to 100 ml of aqueous solution of ammonia used in separate experiments to quench •OH and h^+^ respectively. The experimental conditions were then the same as described in subsection 2.4.

## 4 Result and discussion

### 4.1 Purity and structure analysis

**Figure 2:**
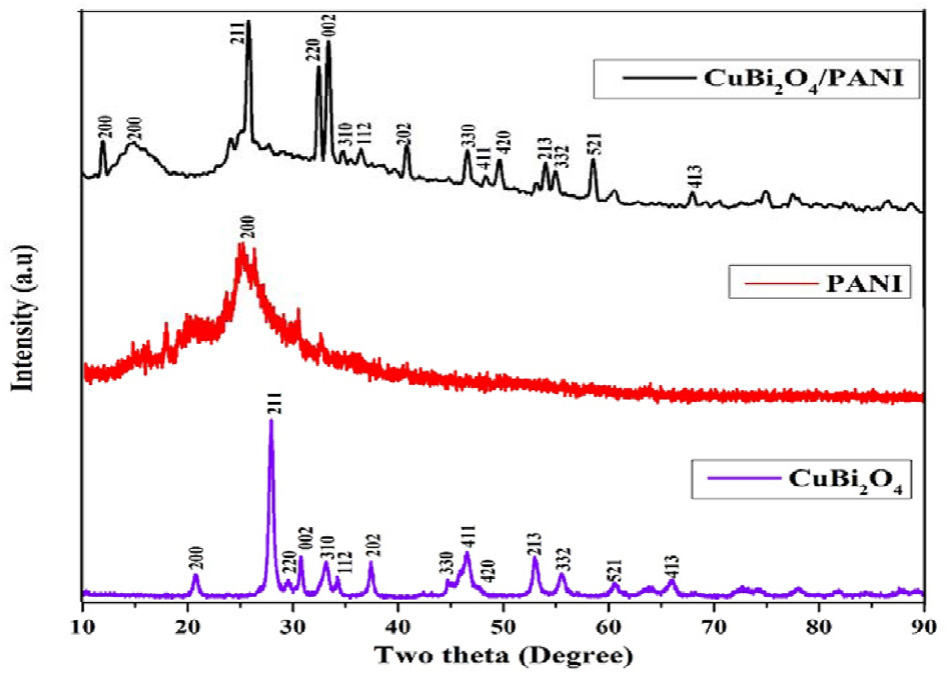
XRD spectra of the as synthesized materials CuBi_2_O_4_, PANI and CuBi_2_O_4_/PANI.

The crystal structure and phase purity of CuBi_2_O_4_, PANI and CuBi_2_O_4_/PANI were confirmed by XRD (Figure 2). Diffraction peaks at 2θ of 20.7°, 27.83°, 29.45°, 30.74°, 33.22°, 34.24°, 37.44°, 44.71°, 46.63°, 47.91°, 53.03°, 55.48°, 60.60° and 65.98° with corresponding *hkl* values (2,0,0), (2,1,1), (2,2,0), (0,0,2), (3,1,0), (1,1,2), (2,0,2), (3,3,0), (4,1,1), (4,2,0), (2,1,3), (3,3,2), (5,2,1) and (4,1,3) show the tetragonal geometry of CuBi_2_O_4_ particles (JCPDS card of CuBi_2_O_4_ (42-0334) (Sharma et al., 2016; Wang et al., 2018). The characteristic hump at 2θ of 25.32° with corresponding *hkl* value of (2,0,0) confirmed the presence of PANI (Mitra et al., 2019). However, in the case of CuBi_2_O_4_/PANI, a clear shift in the peaks toward the lower and higher theta values highlight a mismatch of ionic radii and angle of strain in the composite which confirms the formation of composites (Goransson et al., 2019; Ahmad et al., 2019; Alam et al., 2018). The peak shifts also indicate that the bond formation between PANI and CuBi_2_O_4_ is occurring which was further confirmed with the XPS analysis.

The crystallite size of the particles was calculated with the Scherrer's formula (Equation 2) as follows:

(2) d ​= ​0.9λ/βcosθ

where *d* is the size of the crystallite, *λ* is the x-ray wavelength, *β* is the full width at half maxima and *θ* is the diffraction angle between 10 and 80°. The crystallite size ranged between 18.9, 25.4, and 28.9 nm for CuBi_2_O_4_, PANI and CuBi_2_O_4_/PANI composite, respectively. The presence of the sharp diffraction peaks in CuBi_2_O_4_/PANI and CuBi_2_O_4_ confirmed the crystalline nature of the photocatalyst and the formation of CuBi_2_O_4_/PANI composite.

### 4.2 Morphological characterisation and elemental composition analysis

**Figure 3:**
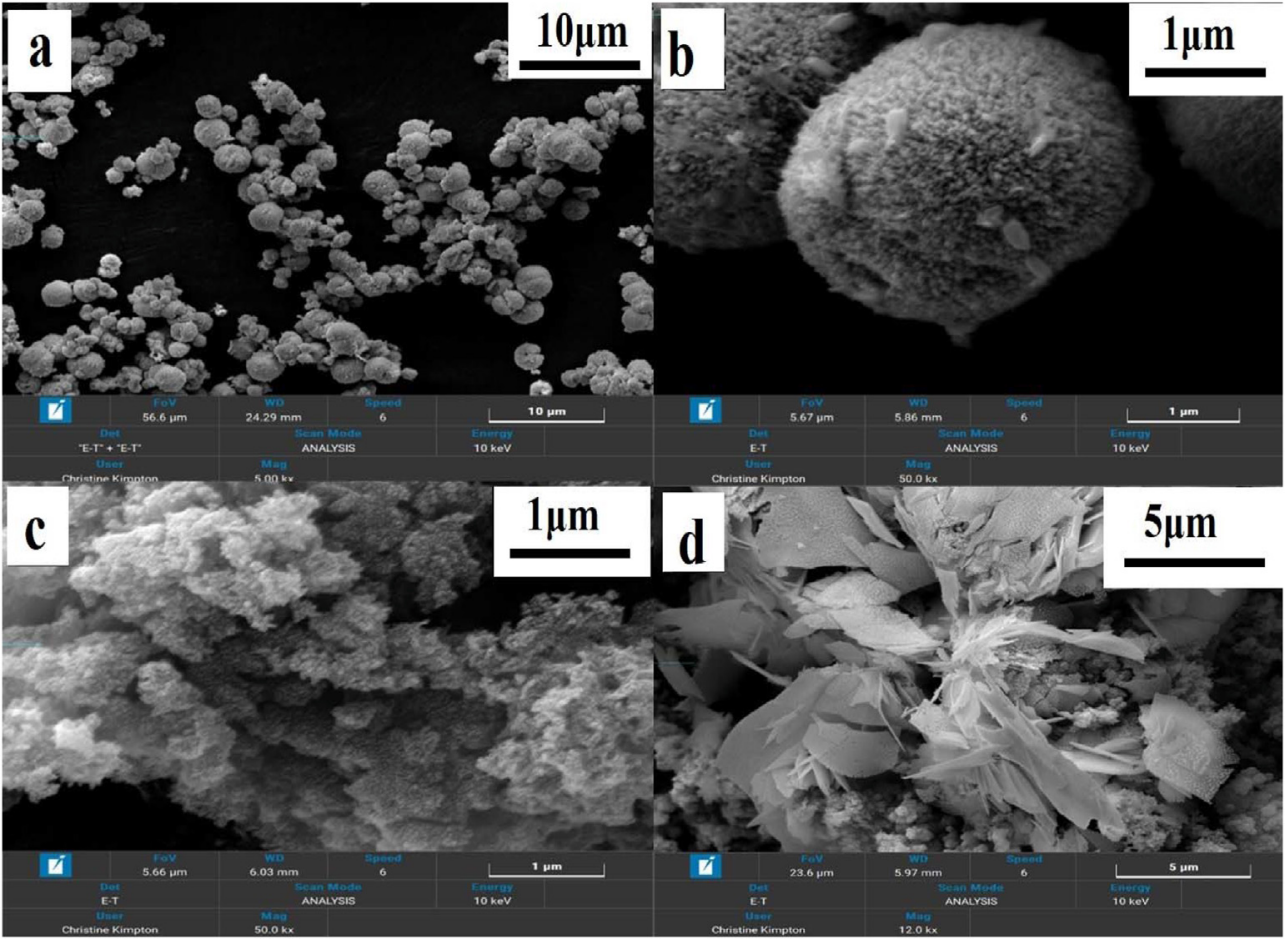
SEM images of as synthesized materials CuBi_2_O_4_ (a, b), PANI (c) and CuBi_2_O_4_/PANI (d).

Surface morphology and shape of CuBi_2_O_4_, PANI and CuBi_2_O_4_/PANI are shown in Figure 3. The hedgehog-like microspheres agglomerated in packed nanorods are the CuBi_2_O_4_ particles (Figure 3, panels a and b). The porous surface of PANI is shown in Figure 3c. The porosity of the surface helps the interaction between ammonia and the photocatalyst. The PANI/CuBi_2_O_4_ composite shape is shown in Figure 3d. The attached microspheres of CuBi_2_O_4_ particles on the surface of the PANI can easily be observed, which implies that the chemical interaction of the PANI and CuBi_2_O_4_ particles was achieved effectively.

EDX image and elemental mapping of the PANI/CuBi_2_O_4_ composite confirmed the presence and distribution of constituent elements (Cu, Bi, O, N and C) and the as shown in Fig.S1 (Supporting Information).

**Figure 4:**
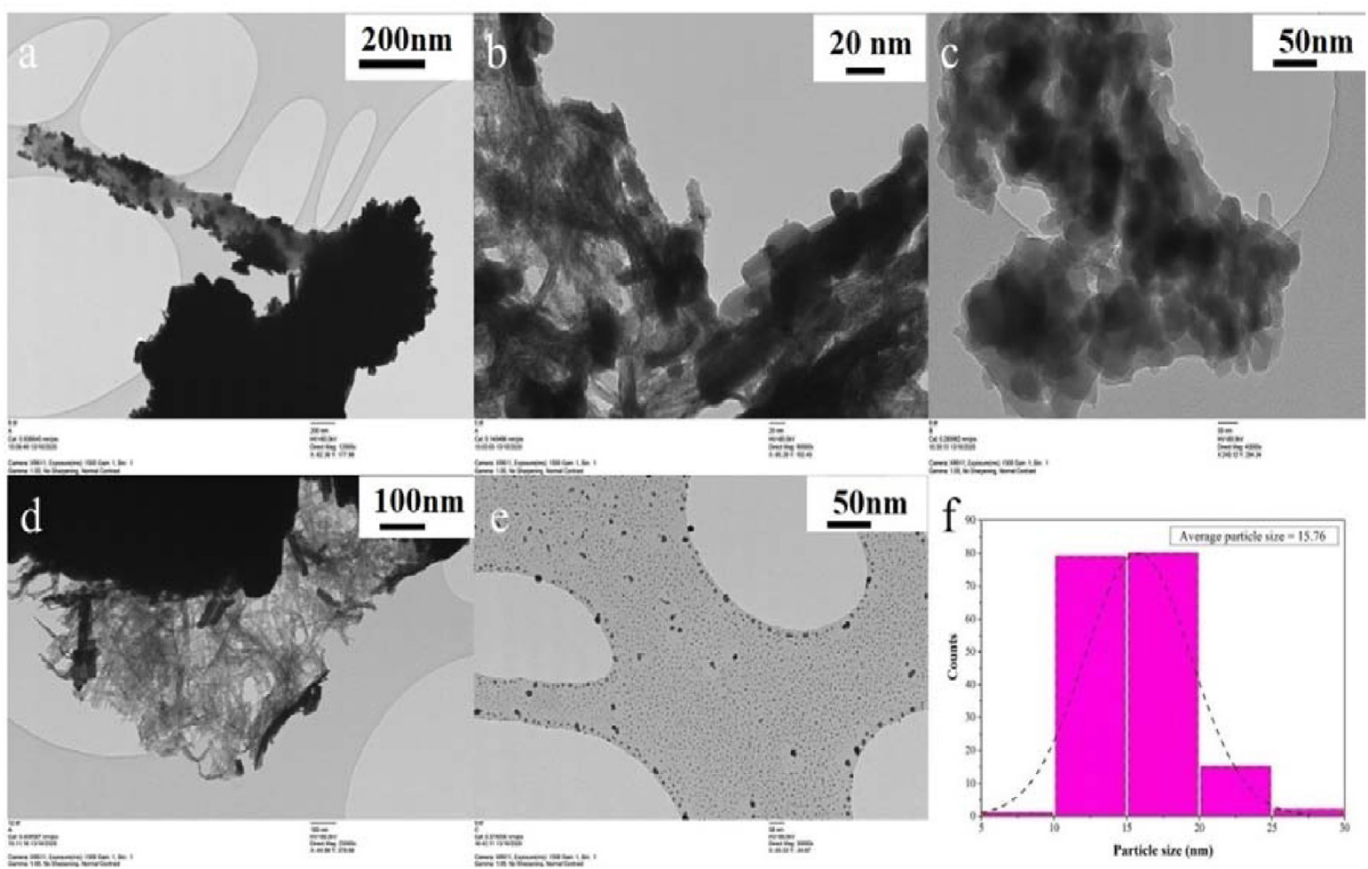
TEM image of CuBi_2_O_4_ (a, b), PANI (c) and CuBi_2_O_4_/PANI (d, e) and particle size distribution (f).

TEM images of the photocatalysts are presented in Figure 4; rod shaped CuBi_2_O_4_ can be seen in Figure 4 panels and b. Panels d and e in Figure 4 show the surface of the PANI and the contact between the PANI and CuBi_2_O_4_. TEM images of the CuBi_2_O_4_ nanorods can also be correlated to the SEM images showing the agglomerated nanorods of the CuBi_2_O_4_ particles (Wang et al., 2015). The averaged particles size of the PANI, CuBi_2_O_4_ and CuBi_2_O_4_/PANI was 29.03 ± 1.2 nm, 21 ± 1.0 nm and 15.76 ± 1.5 nm, respectively.

### 4.3 Chemical state of CuBi_2_O_4_ and PANI/CuBi_2_O_4_ photocatalysts

**Figure 5:**
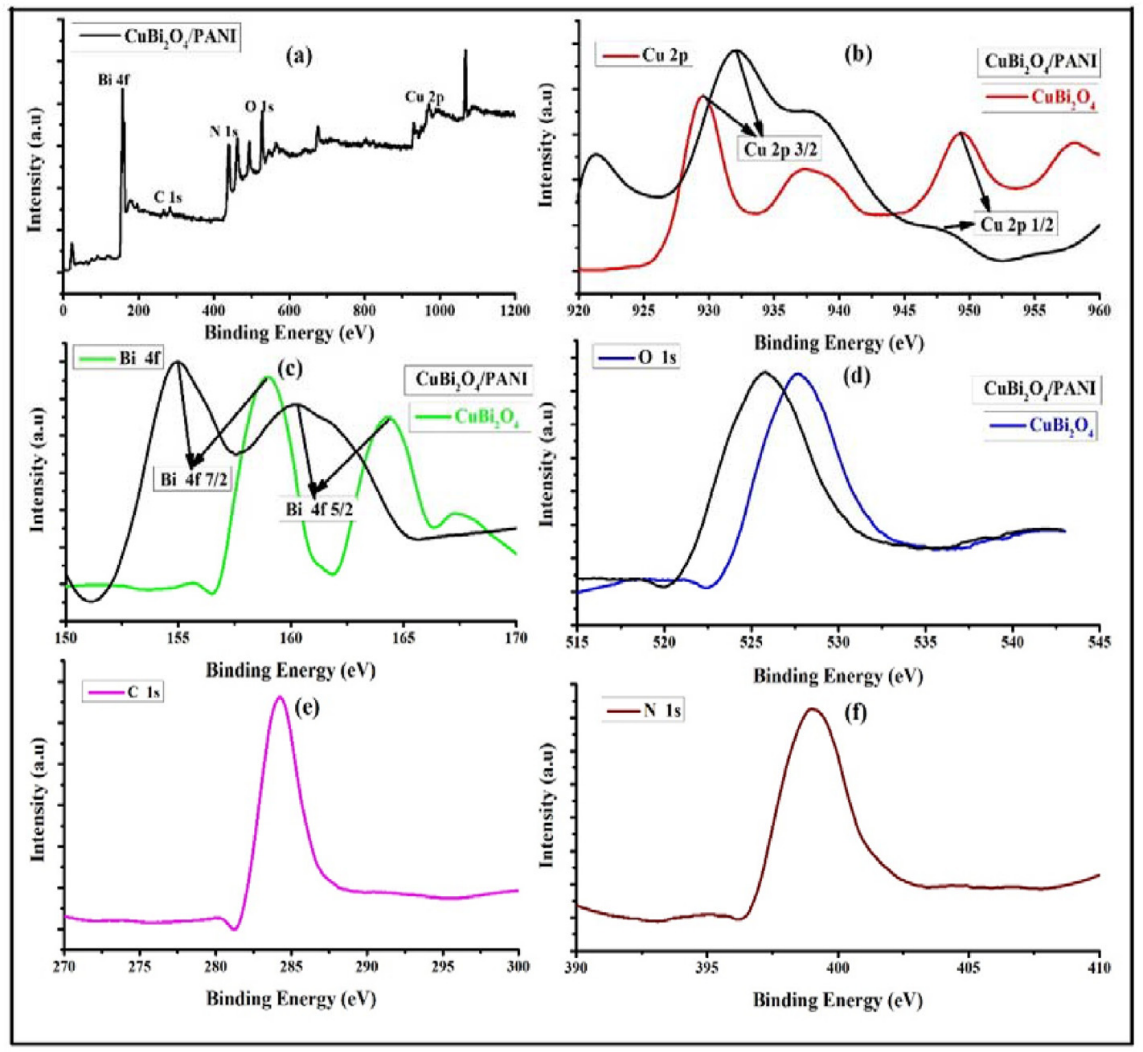
X ray photon survey spectra of (a) CuBi_2_O_4_/PANI composites (b) Cu 2p (c) Bi 4f (d) O 1s (e) C 1s and (f) N 1s recorded at room temperature.

The XPS analysis was performed to characterise the chemical charge states, the surface elemental composition of the CuBi_2_O_4_ and CuBi_2_O_4_/PANI photocatalysts and to identify possible impurities (Figure 5). The photoelectron peaks of Cu, Bi, O, C, and N elements were clearly identified. The binding energies of the elements are shown in Table 1.

**Table: 1:** Representative elements with corresponding binding energies.

Elemental states	Binding Energy (eV)
(Cu 2p_3/2_) (Cu 2p_1/2_)	929.24 949.44
(Bi 4f _7/2_) (Bi 4f _5/2_)	158.93 164.23
(O 1s)	527.63
(C 1s)	284.19
(N 1s)	399.1

In pure CuBi_2_O_4_, the binding energy peaks of (Cu 2p_3/2_) and (Cu 2p_1/2_) are located at 929.24 and 949.44 eV; (Bi 4f _7/2_) and (Bi 4f _5/2_) peaks are located at 158.93 and 164.23 eV while (O 1s), owing to lattice oxygen of CuBi_2_O_4_, is located at 527.63 eV. These peaks are slightly shifted in PANI/CuBi_2_O_4_ which confirmed the heterojunction between PANI and CuBi_2_O_4_. Our measured values are in good agreement with those reported by Shi et al. (2017) who reported 934 and 954 eV for Cu 2p_3/2_ and Cu 2p_1/2_, respectively and 158.5 and 164 eV for (Bi 4f _7/2_) and (Bi 4f _5/2_). For PANI-CuBi_2_O_4_, the binding energy peaks (Cu 2p_3/2_ -932.3 eV), Cu 2p_1/2_-948.4 eV), (Bi 4f _7/2_- 154.9 eV), (Bi 4f _5/2_- 166.4 eV), (O 1s- 525.51 eV) (C 1s-284.19 eV) and (N 1s-399.1 eV) were identified which indicates the chemical interaction between PANI and CuBi_2_O_4_ (Bano et al., 2018). The C 1s spectrum at 284.19 eV confirmed the sp^2^ hybridised carbon and the N 1s spectrum at 399.1 eV confirmed the N-H bond in the PANI (Guo et al., 2017).

### 4.4 BET analysis

**Figure 6:**
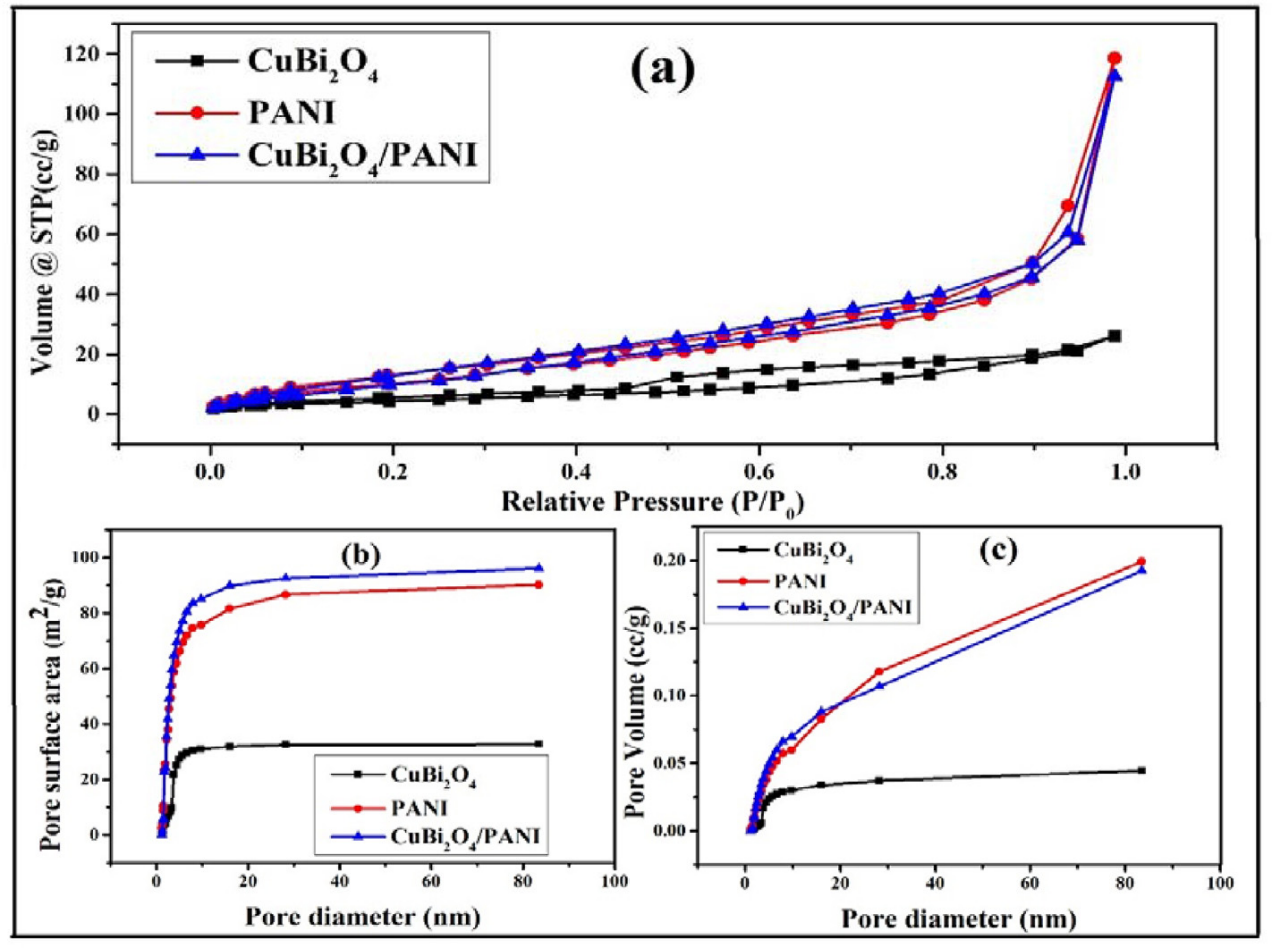
(a) BET isotherm, (b) pore surface area and (c) pore volume.

The specific surface areas of the catalysts were estimated by the Brunauer–Emmett–Teller (BET) method from the nitrogen adsorption-desorption isotherm. The BET isotherms pore volumes and pore surface areas are shown in Figure 6a. Surface areas, pore diameters and pore volumes of the photocatalysts are summarised in Table 2. CuBi_2_O_4_/PANI exhibits higher surface area than either pure PANI or CuBi_2_O_4_; this was due to the doping of CuBi_2_O_4_ into the polymer. The surface area increases as particle size decreases. The higher surface area of CuBi_2_O_4_/PANI is due to the porous surface of polyaniline in which CuBi_2_O_4_ particles are settled down therefore reduces the size of PANI/CuBi_2_O_4_ (Dubois et al., 2011).

**Table: 2:** Surface area, pore diameter and pore volume of photocatalyst.

Catalyst	BET surface area, (m^2^g^−1^)	BJH Pore diameter (nm)	Total BJH Pore volume, (cm^3^g^−1^)
CuBi_2_O_4_	16.46	3.84	0.045
PANI	42.28	1.54	0.20
CuBi_2_O_4_/PANI	45.51	1.50	0.19

### 4.5 Bandgap energy analysis and calculation of edge band potential

**Figure 7:**
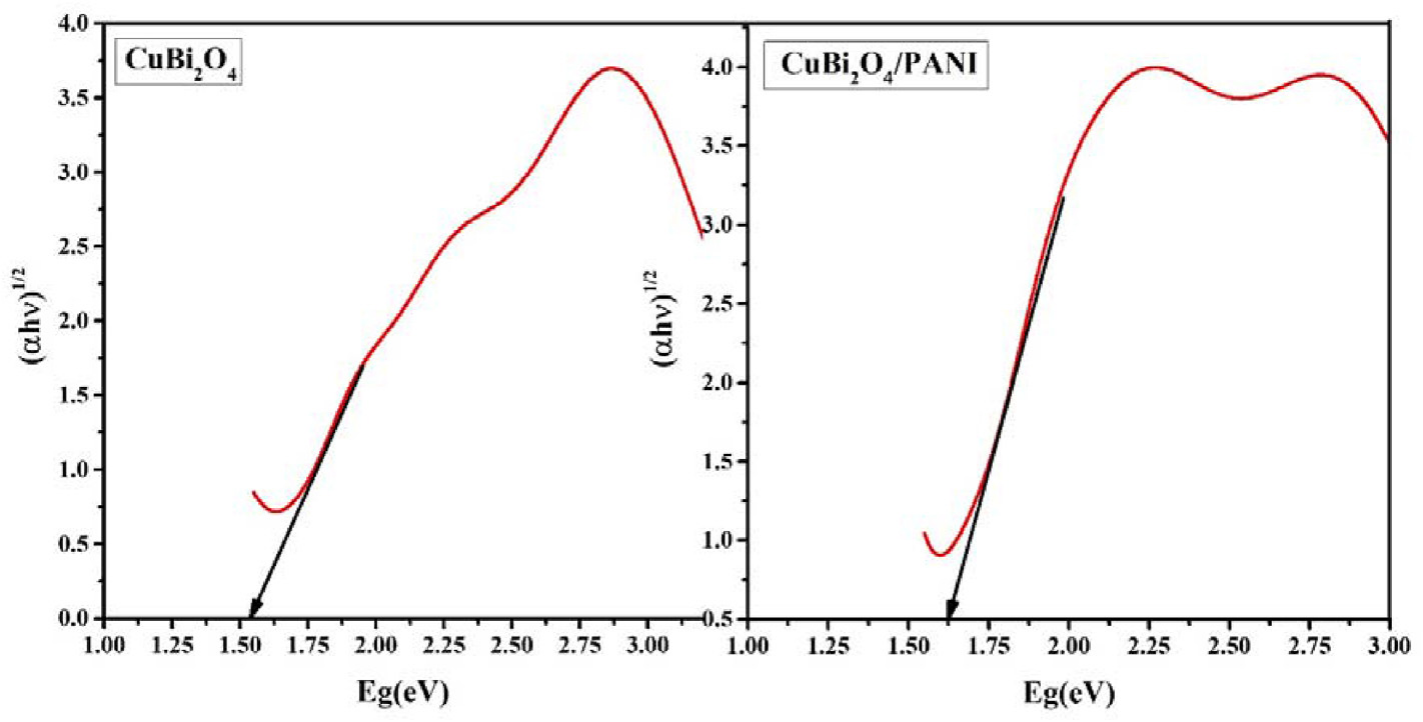
Tauc plot of CuBi_2_O_4_ and CuBi_2_O_4_/PANI composites.

To elucidate the photocatalytic mechanism, bandgap energy and edge band potential of the conduction band and valence band were investigated by the Tauc plot (Figure 7) based on the Kubelka Munk Function formula (Equation 3):

(3) $$\left(\text{hv.}\alpha \right)\text{ ​}=\text{ ​}{\left(\text{Ahv}-\text{Eg}\right)}^{\text{n/}2}$$

where *α* is proportional to F(R), which is Kubelka Munk function, *ν* is the frequency, *A* is the proportionality constant and *Eg* is the bandgap energy. Now it can be written as shown in Eq. (4),

(4) $$\left\{(\text{hv.F}(\text{R})\right\}\text{ ​}=\text{ ​}{\left(\text{Ahv}-\text{Eg}\right)}^{\text{n/}2}$$

The value of *n* is determined by n = 1 or 4 for direct and indirect allowed transition, respectively. The indirect transition (n = 4) has been considered to calculate the bandgap of PANI and CuBi_2_O_4_.

As shown in Figure 7, the respective bandgap energies for CuBi_2_O_4_ and CuBi_2_O_4_/PANI were 1.5 and 1.7 eV (Arai et al., 2007). The bandgap energy of the pure PANI was 2.1 eV as previously calculated by Ahmad et al., 2019a, Ahmad et al., 2019b.

With the help of these bandgap energy values, conduction band (CB) and valence band (VB) potentials of the photocatalysts were calculate with Eqs. (5) and (6):

(5) $${\text{E}}_{\text{VB}}\text{ ​}=\text{ ​X}-\text{ ​}{\text{E}}^{\text{c}}\text{ ​}{+0.5\text{E}}_{\text{g}}$$

(6) $${\text{E}}_{\text{CB}}\text{ ​}=\text{ ​}{\text{E}}_{\text{VB}}{-\text{E}}_{\text{g}}$$

where *E*_VB_ and *E*_CB_ are the VB and CB edge potential of the photocatalyst, *E*^*c*^ is the energy of free electrons on hydrogen's scale (i.e. 4.5 eV) and X is the geometric mean of the electronegativity of the constituent atoms of the photocatalyst. The respective conduction band potentials for PANI and CuBi_2_O_4_ were -1.38 and -0.61eV vs normal hydrogen electrode (NHE); the valence band potential of the PANI and CuBi_2_O_4_ were 0.8 and 1.13 eV, respectively.

### 4.6 Photocatalytic activity and extent of ammonia degradation

The photodegradation activity of the photocatalysts was checked by degrading ammonia in water. The rate constants of the photocatalytic reaction were fitted to zero-order kinetics. Table 3 list constant rates and removal extents (Table 3). The plots of Ln C vs time and Concentration vs time are shown in Figures S2 and S3 (Supporting information).

**Table: 3:** Table representing the rate constants and degradation efficiencies of the photocatalyst.

Photocatalysts	Rate constants (min^−1^)	Degradation efficiency (%)
CuBi_2_O_4_	0.6475 × 10^−2^	70
PANI	0.8896 × 10^−2^	78
CuBi_2_O_4_/PANI	1.28 × 10^−2^	96

**Figure 8:**
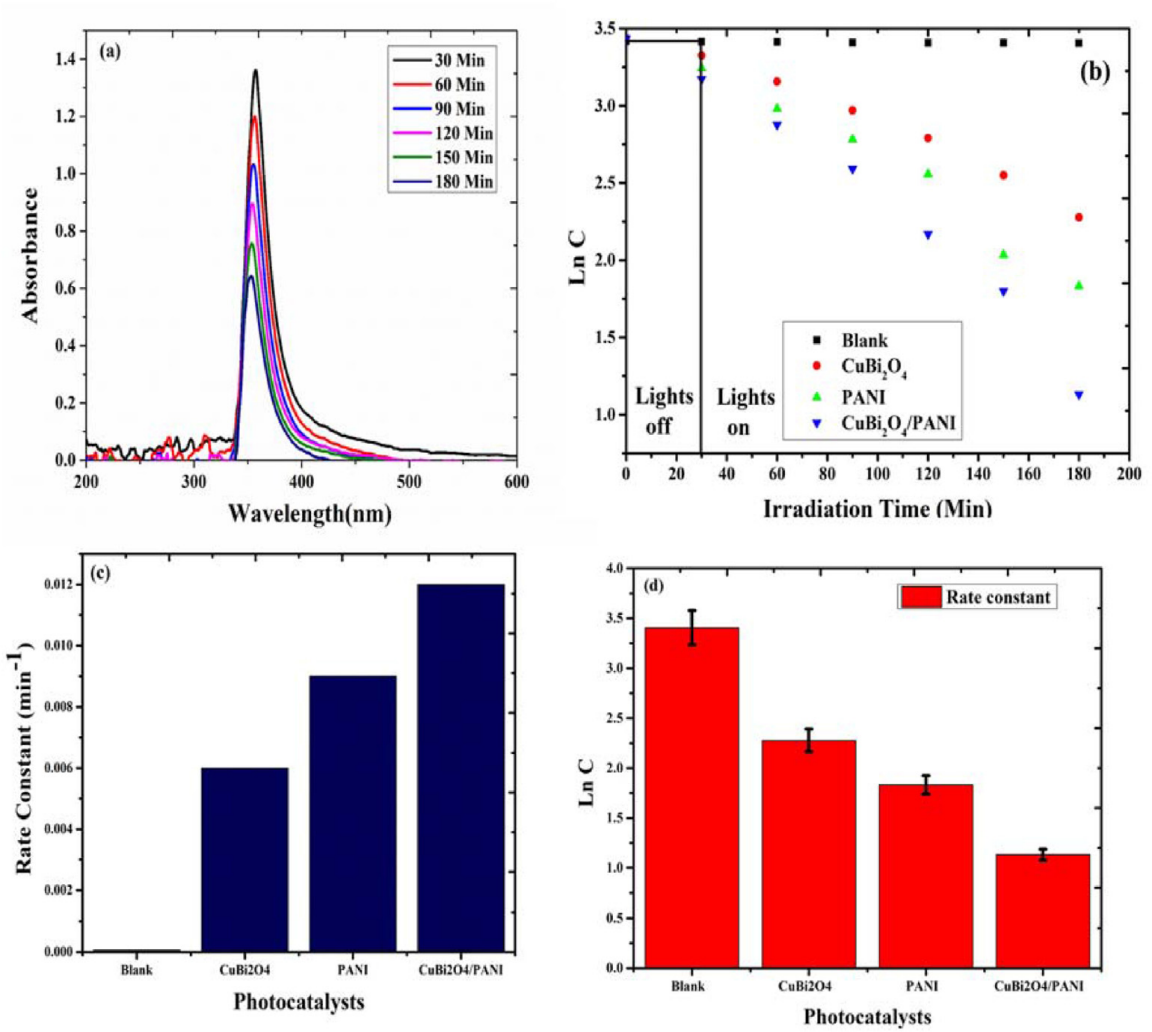
(a) UV-visible spectra of the photodegraded ammonia in water samples (b) Kinetics of photodegradation(c) rate constant of various photocatalyst against ammonia and (d) Standard deviation value of the kinetics of photodegradation from three independent replicates.

The photocatalytic experiments for the degradation of ammonia was repeated three times to ensure the applicability of the photocatalyst. The kinetics of ammonia photodegradation along the UV-visible spectra of the photodegraded ammonia in water samples and the rate constant of photocatalysts are shown in Figure 8.

**Figure 9:**
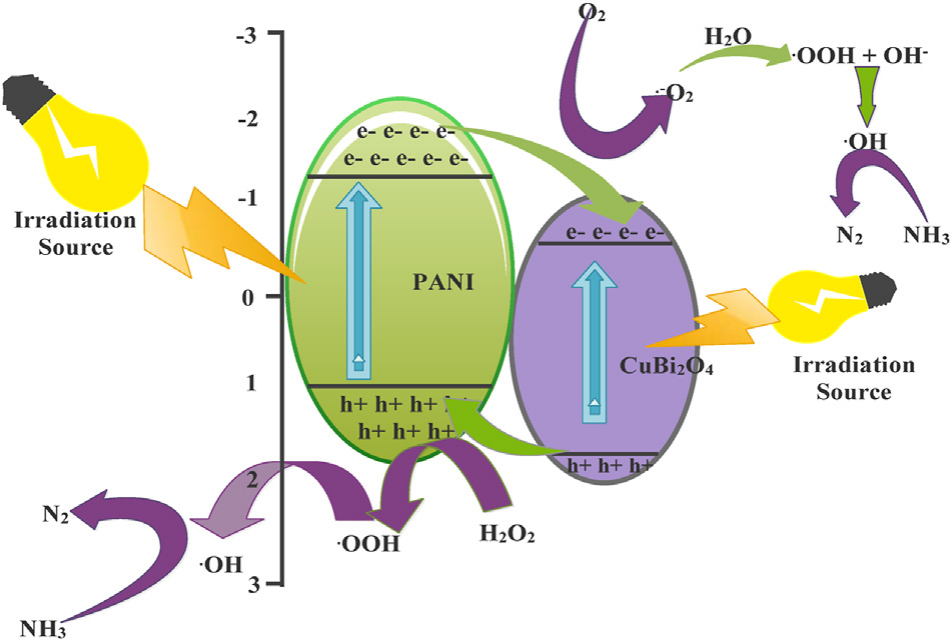
Proposed photocatalytic mechanism for the degradation of ammonia.

Photocatalytic process started with the irradiation of LED lights. Upon irradiation, PANI was excited and generates electron-hole pairs in the highest occupied molecular orbital (HOMO) and the lowest unoccupied molecular orbital (LUMO), respectively. When CuBi_2_O_4_ was irradiated with LED, it absorbed the photons and got excited and then the electrons travelled to the conduction band (CB) leaving holes in the valence band (VB) as shown in Figure 9.

The electrons in the CB of the CuBi_2_O_4_ were in an unstable state and tended to occupy stable state, thus recombining with the holes. In this case, there were fewer electrons available to form ROS through redox reaction. Hence, the ammonia degradation by photocatalytic activity was quite slow; similarly, the PANI photodegradation efficiency was only of 78% for ammonia removal and this was due to higher recombination rates of electron and holes. In contrast, the combined CuBi_2_O_4_/PANI catalyst had the highest photocatalytic activity achieving over 96% removal of ammonia within 180 min. The formation of composites of CuBi_2_O_4_ with PANI reduced the recombination of electrons and holes and contributed to the formation of hydroxyl radical. The higher edge band potential of the PANI (−1.38 eV) compared to CuBi_2_O_4_ (−0.61 eV) allowed the electrons of the LUMO of PANI to flow easily toward the CuBi_2_O_4_ particles scattered on the surface of PANI. In this way, the LUMO excited electrons of PANI, which are supposed to acquire a stable state, were combined with the excited electrons of the CuBi_2_O_4_ and thus suppress the recombination of electron-hole pairs of the PANI. As a result of the surface plasmon resonance (SPR) effect together with the electrons transferred from PANI, the photo excited electrons of CuBi_2_O_4_ convert the molecular tosuperoxide˙O_2_¯. These ˙O_2_¯ molecules will then react with H_2_O molecules to form •OOH and OH^−^ and subsequently form H_2_O_2_. The generated holes in the HOMO of PANI then reacted with the H_2_O_2_ molecule and produced ^**•**^OH radicals. The ROS generated during the redox process (h^+^ and ^**˙**^OH) contributed to the conversion of ammonia into nitrogen. The following reactions (7-16) describe the mechanism of photodegradation:

(7) $$\text{PANI ​}+\text{ ​}{\text{CuBi}}_{2}{\text{O}}_{4}\text{ ​}+\text{ ​h}u\to {\text{PANI/CuBi}}_{2}{\text{O}}_{4}\text{ ​}\left({\text{h}}^{+}\text{, ​}{\text{e}}^{-}\right)$$

(8) $$\text{PANI ​}\left({\text{e}}^{-}\right)\text{ ​}+\text{ ​}{\text{O}}_{2}\to \cdot {\text{O}}_{2}^{-}$$

(9) $${\text{H}}_{2}\text{O ​}+\text{ ​}\cdot {\text{O}}_{2-}\to \cdot \text{OOH ​}+\text{ ​}{\text{OH}}^{-}$$

(10) $$\cdot \text{OOH ​}+\text{ ​}{\text{H}}_{2}\text{O ​}\to \text{ ​}\cdot \text{OH ​}+\text{ ​}{\text{H}}_{2}{\text{O}}_{2}$$

(11) $${\text{H}}_{2}{\text{O}}_{2}\text{ ​}+\text{ ​}{\text{h}}^{+}\text{ ​}\to \text{ ​}\cdot \text{OOH ​}+\text{ ​}{\text{H}}^{+}$$

(12) $${\text{H}}_{2}{\text{O}}_{2}\text{ ​}+\text{ ​}\cdot \text{OOH ​}\to \text{ ​}\cdot \text{OH ​}+\text{ ​}{\text{H}}_{2}\text{O ​}+\text{ ​}{\text{O}}_{2}$$

The · OH generated react with ammonia to produce nitrogen

(13) $${\text{NH}}_{3}+\text{ ​}\cdot \text{OH ​}\to \text{ ​}{\text{NH}}_{2}\text{ ​}+\text{ ​}{\text{H}}_{2}\text{O}$$

(14) $${\text{NH}}_{2}+\text{ ​}\cdot \text{OH ​}\to \text{ ​NH ​}+\text{ ​}{\text{H}}_{2}\text{O}$$

(15) $$\text{NH}+\text{ ​}\cdot \text{OH ​}\to \text{ ​N ​}+\text{ ​}{\text{H}}_{2}\text{O}$$

(16) $${\text{NH}}_{3}+\text{ ​}{\text{h}}^{+}\text{ ​}\to \text{ ​}{\text{N}}_{2}\text{ ​}+\text{ ​}{\text{H}}^{+}$$

In the mechanistic approach for the degradation of ammonia, CuBi_2_O_4_ particles facilitate the efficient separation of charge carriers, which increases lifetime and enhances degradation efficiency. The photocatalytic activity of the novel CuBi_2_O_4_/PANI composite is high compared to those reported in literature for ammonia (Table 4).

**Table: 4:** Comparison of photodegradation efficiency of several photocatalysts for ammonia removal.

S.No	Photocatalyst	Ammonia (mg/l)	Photocatalyst (g)	Removal Efficiency (%)	Irradiation time (min)	Irradiation source	Reference
1	Ag_3_PO_4_-CaO	340	1.25	70	240	Visible lamp 53 mW/cm^2^	Shavisi and Sharifnia, 2018
2	Zinc Ferrite/Activated Carbon	100	1.5	90	180	High pressure mercury lamp 300 W	Ye et al., (2019)
3	Cu/ZnO/rGO	50	0.2	83	120	High pressure mercury lamp 125 W	He et al., (2018)
4	TiO_2_-ZnO/LECA	400	25	95	180	High pressure mercury lamp 125 W	Mohammadi et al., (2016)
5	UiO-66(Ti)-Fe_3_O_4_-WO_3_	30	0.125	92	60	LEDs (14.4 W/m, 12 v)	Bahmani et al., (2020)
6	TiO_2_ -Perlite	170	11.7	68	180	125 W Hg lamp	Shavisi et al., (2014)
7	α-MnO_2_/N-Doped Graphene	100	0.1	93	480	NIR light irradiation	Liu et al., (2018)
8	Biochar carbon-doped TiO_2_/CuO	100	0.03	99	120	25 W UV lamp	Peng et al., (2019)
9	TiO_2_/LECA	975	125	96	4320	Solar Light	Shavisi et al., (2014)
10	PANI-CuBi_2_O_4_	30	0.1	96	180	LEDs (15 W)	Present study

### 4.7 Effect of pH on degradation of ammonia and photoluminescence studies

**Figure 10:**
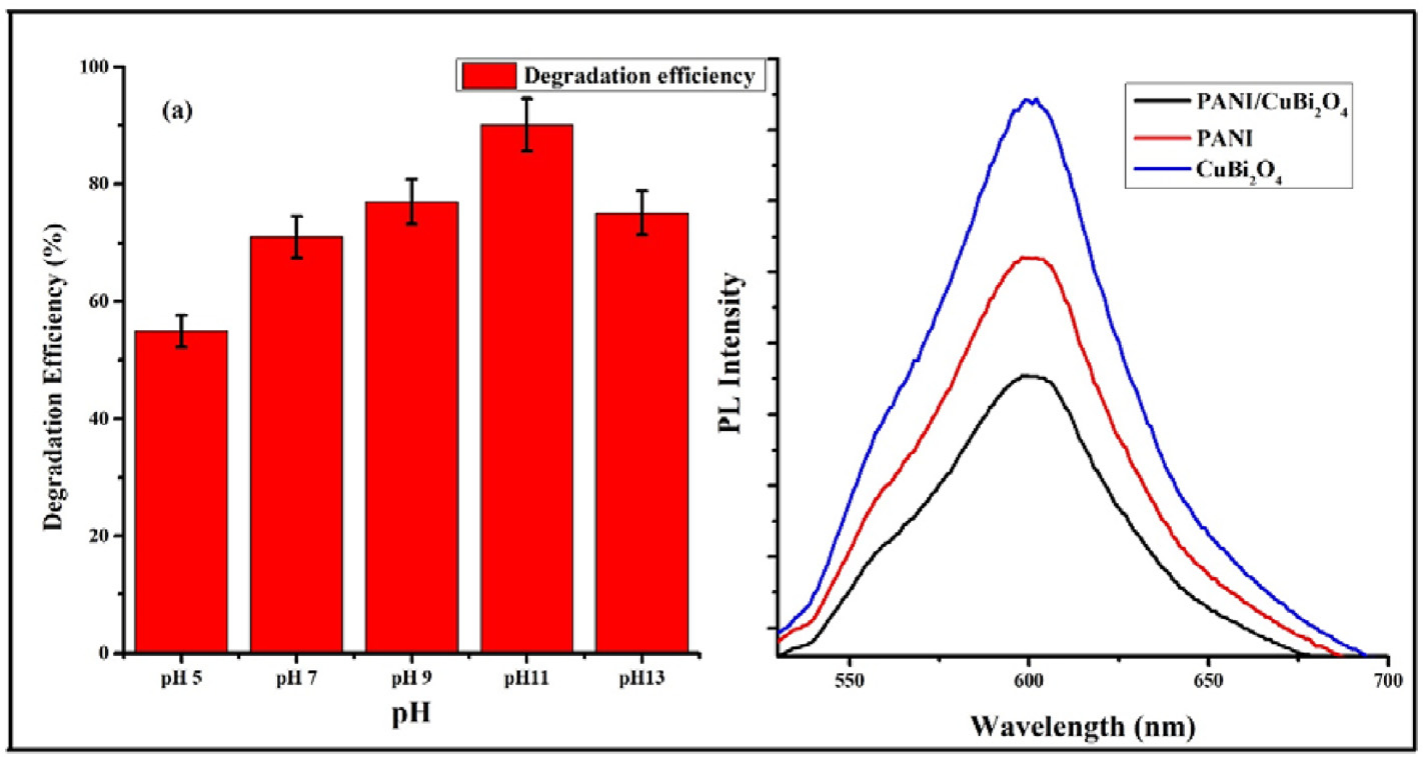
Effect of pH on the photodegradation of ammonia (a) and PL spectra of the photocatalyst to check the recombination behaviour of electron and holes (b).

Photodegradation efficiency is significantly influenced by pH (Shavisi et al., 2014; Altomare et al., 2012). The optimum pH was 11 for the degradation of ammonia in water. At basic pH, the formation of hydroxide ion and subsequently into hydroxyl radical favours the photodegradation of ammonia as shown in Figure 10a. Degradation efficiency increased from 55 to 90% up to pH 11 and decreased to 75% at pH 13. This was due to the competitive adsorption of hydroxyl radical on the surface of the photocatalyst which lowers the degradation efficiency.

Further, to check the recombination behaviour of the photogenerated electron and hole pairs, fluorescence spectroscopy was used. The recombination rate of electrons and holes are directly proportional to photocatalytic activity (Bano et al., 2019; Shafi et al., 2019). Figure 10b shows photoluminescence (PL) intensity of CuBi_2_O_4_, PANI and CuBi_2_O_4_/PANI. Higher PL intensities for PANI and CuBi_2_O_4_ indicate higher recombination rates of photogenerated electrons and holes, which translates to a lower photocatalytic activity. Conversely, the lower PL intensity observed for CuBi_2_O_4_/PANI was due to a lower recombination of electrons and holes that led to higher photocatalytic activity.

### 4.8 Tracking of the reactive species and durability of photocatalyst

**Figure 11:**
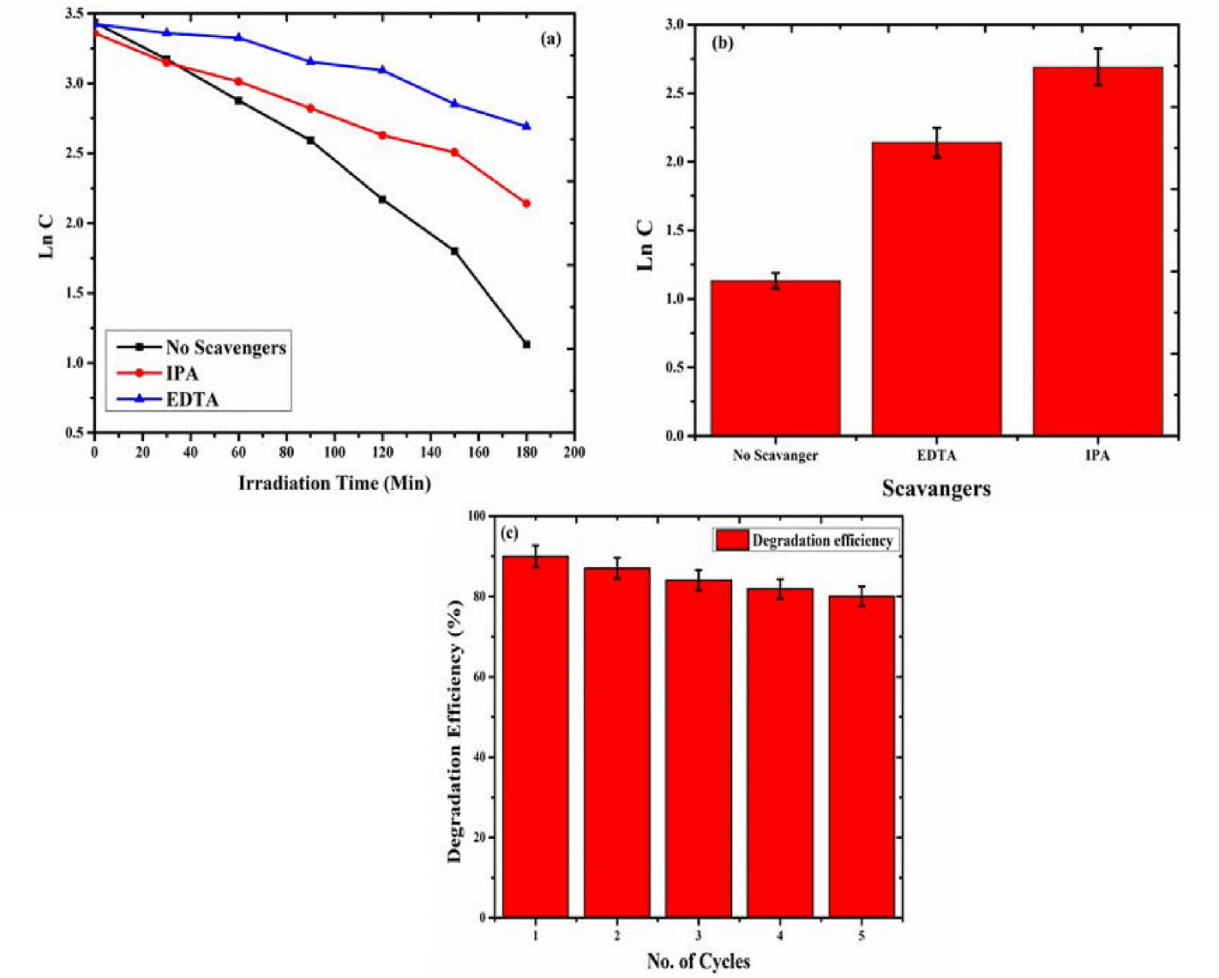
(a) Trapping experiments by holes and hydroxyl radicals (b) standard deviation of the trapping experiment and (c) recycling experiment of CuBi_2_O_4_/PANI photocatalyst.

IPA and EDTA were introduced to the photocatalytic degradation of ammonia with CuBi_2_O_4_/PANI to observe the effect •OH and h^+^ respectively. The rate constant of CuBi_2_O_4_/PANI declined when the scavengers were added (Fig. 11a and b). Both holes and hydroxyl radicals can contribute to ammonia degradation. Nevertheless, holes are the primary reactive species involved in such process, as holes produce hydroxyl radicals by oxidation of water molecules.

To assess the durability of CuBi_2_O_4_/PANI, the same photocatalyst was used for five consecutive times. Hence, the photocatalyst was recovered after each experiment, washed with distilled water and acetone, and dried for 12 h at 60 °C. As Figure 11c shows, the photocatalytic efficiency decreased by less than 2% after each experiment. Overall, the efficiency was found to be <10% lower after the five consecutive tests. The decreased in the percentage removal was attributed to loss of photocatalyst particles after each use.

## 5 Conclusions

While PANI and CuBi_2_O_4_ showed good performance, the CuBi_2_O_4_/PANI composite provided better activity towards the photodegradation of ammonia in water. The removal extent for CuBi_2_O_4_/PANI was 96% whereas the respective values for CuBi_2_O_4_ and PANI were 70% and 78%. CuBi_2_O_4_/PANI outperformed the other materials owing to a higher electron and holes separation, lower recombination rates of electron and holes, and greater surface area. Both holes and hydroxyl radicals were accountable for the degradation of ammonia, but holes were the primary reactive species. CuBi_2_O_4_/PANI also exhibited reusability properties without compromising its activity. Based on the results and generation of reactive species responsible, it can be concluded that CuBi_2_O_4_/PANI is a promising photocatalyst for the treatment ammonia affluent.

## Declarations

### Author contribution statement

Nafees Ahmad: Conceived and designed the experiments; Performed the experiments; Analyzed and interpreted the data; Wrote the paper.

Jerry Anae: Contributed reagents, materials, analysis tools or data; Wrote the paper.

Mohammad Zain Khan, Suhail Sabir, Pablo Campo & Frederic Coulon: Conceived and designed the experiments; Wrote the paper.

### Funding statement

Nafees Ahmad was supported by Commonwealth Foundation [INCN-2019-35].

### Data availability statement

Data included in article/supp. material/referenced in article.

### Declaration of interests statement

The corresponding author Prof Frederic Coulon is one of the Section Editor of the Environment Section.

### Additional information

Supplementary content related to this article has been published online at https://doi.org/10.1016/j.heliyon.2022.e10210.

## Unlisted reference

Shaveisi et al. 2018
