# Supplementary material for: A novel CuBi2O4/polyaniline composite as an efficient photocatalyst for ammonia degradation
Source: Heliyon. 2022 Aug 18;8(8):e10210. doi: 10.1016/j.heliyon.2022.e10210 (PMC9420373; doi:10.1016/j.heliyon.2022.e10210)
Supplement: Supporting Information [file mmc1.docx]

**Supporting Information**

**A novel CuBi_2_O_4_/polyaniline composite as an efficient photocatalyst for ammonia degradation**

Nafees Ahmad^1,3^, Jerry Anae^1^, Mohammad Zain Khan^2^, Suhail Sabir^2^, Pablo Campo^1^, Frederic Coulon^1*^

^1^ School of Water, Energy and Environment, Cranfield University, Cranfield, MK 43 0AL, UK

^2^Environmental Research Laboratory, Department of Chemistry, Aligarh Muslim University, Aligarh, India, 202002

^3^Department of Chemistry, Integral University, Lucknow, India, 226026

**
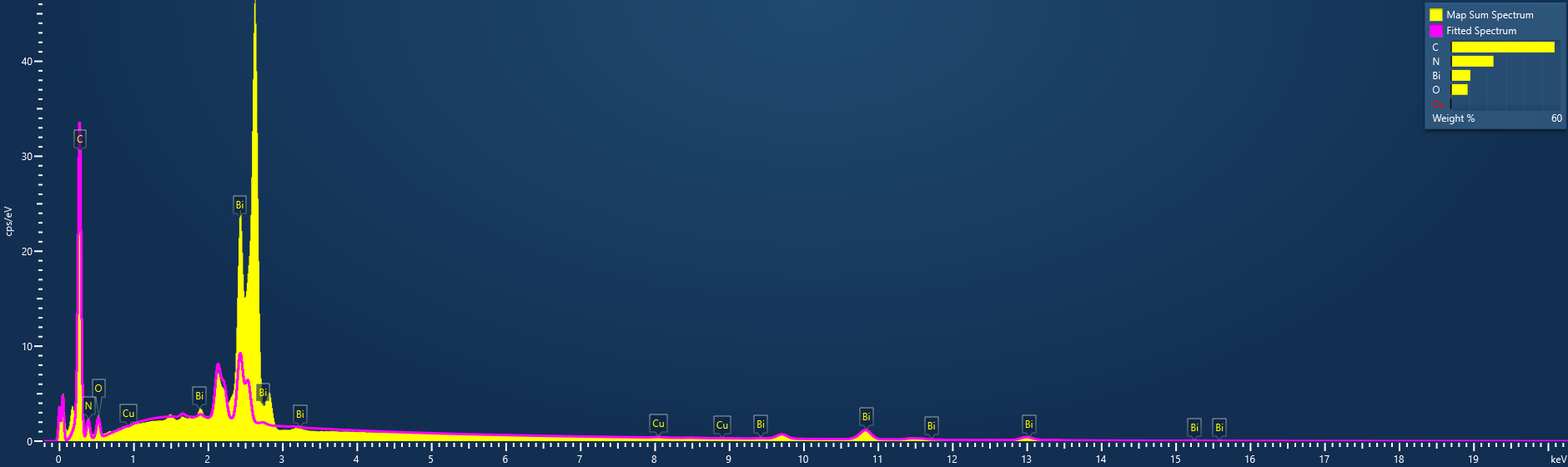
**

**
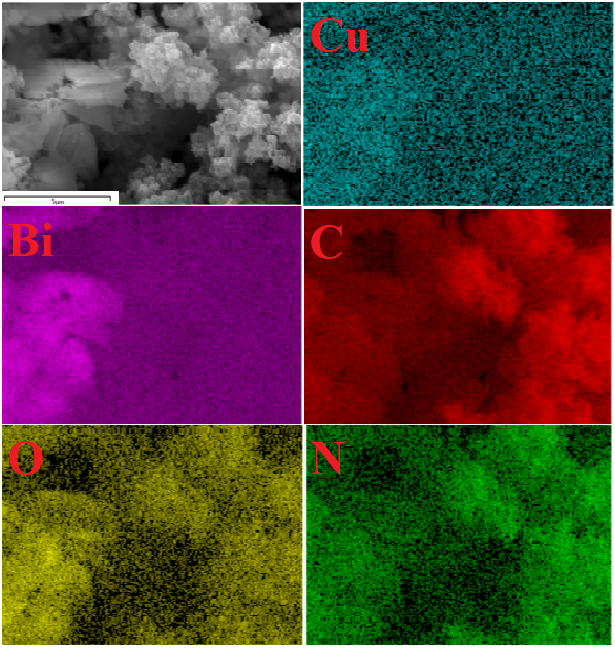
**

**Fig. S1.** EDX image of CuBi_2_O_4_/PANI (top) and elemental map of the elements present in the materials (coloured panels)


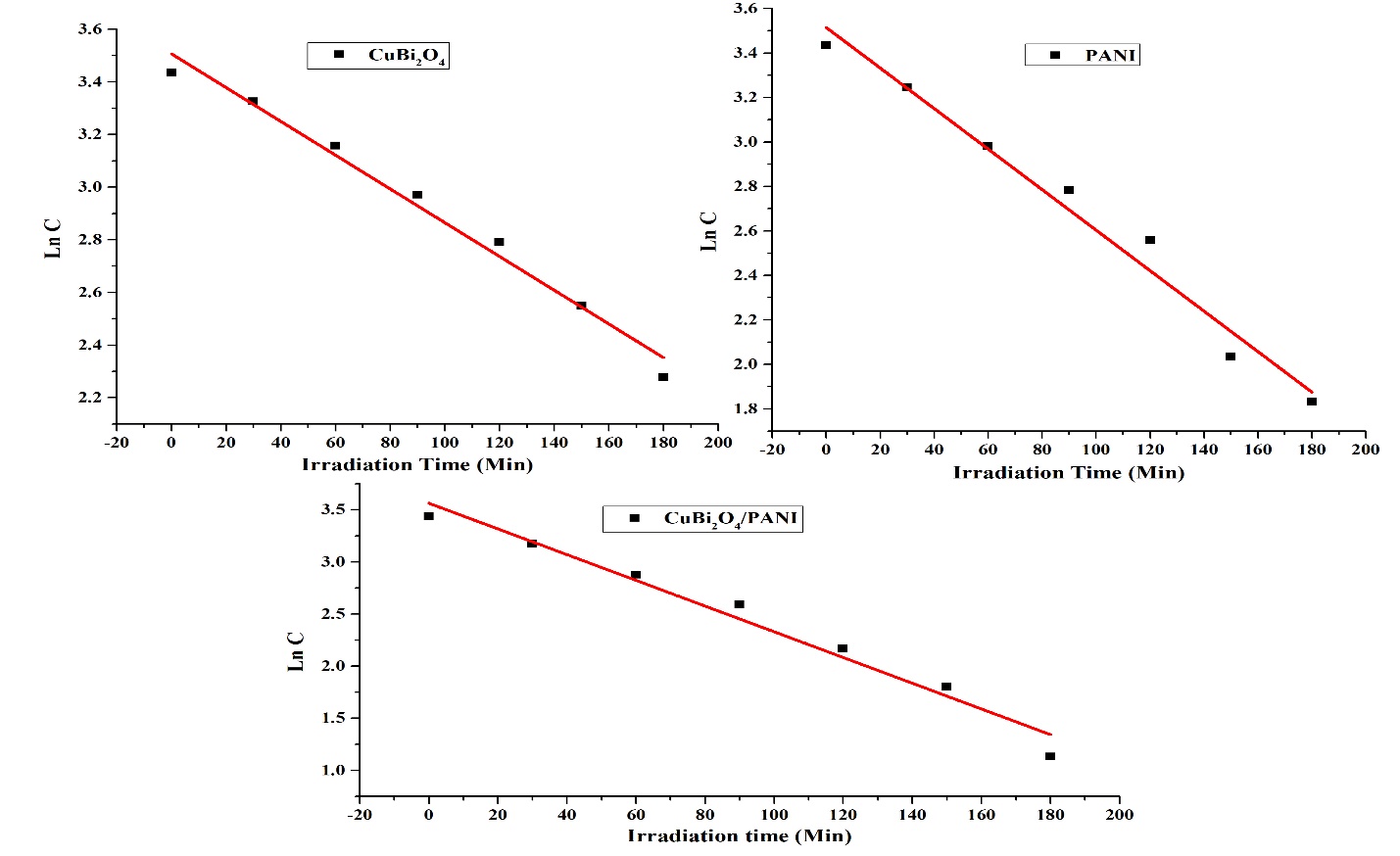


**Fig. S2**. Plots of LnC vs time shows the correlation of the data by regression analysis


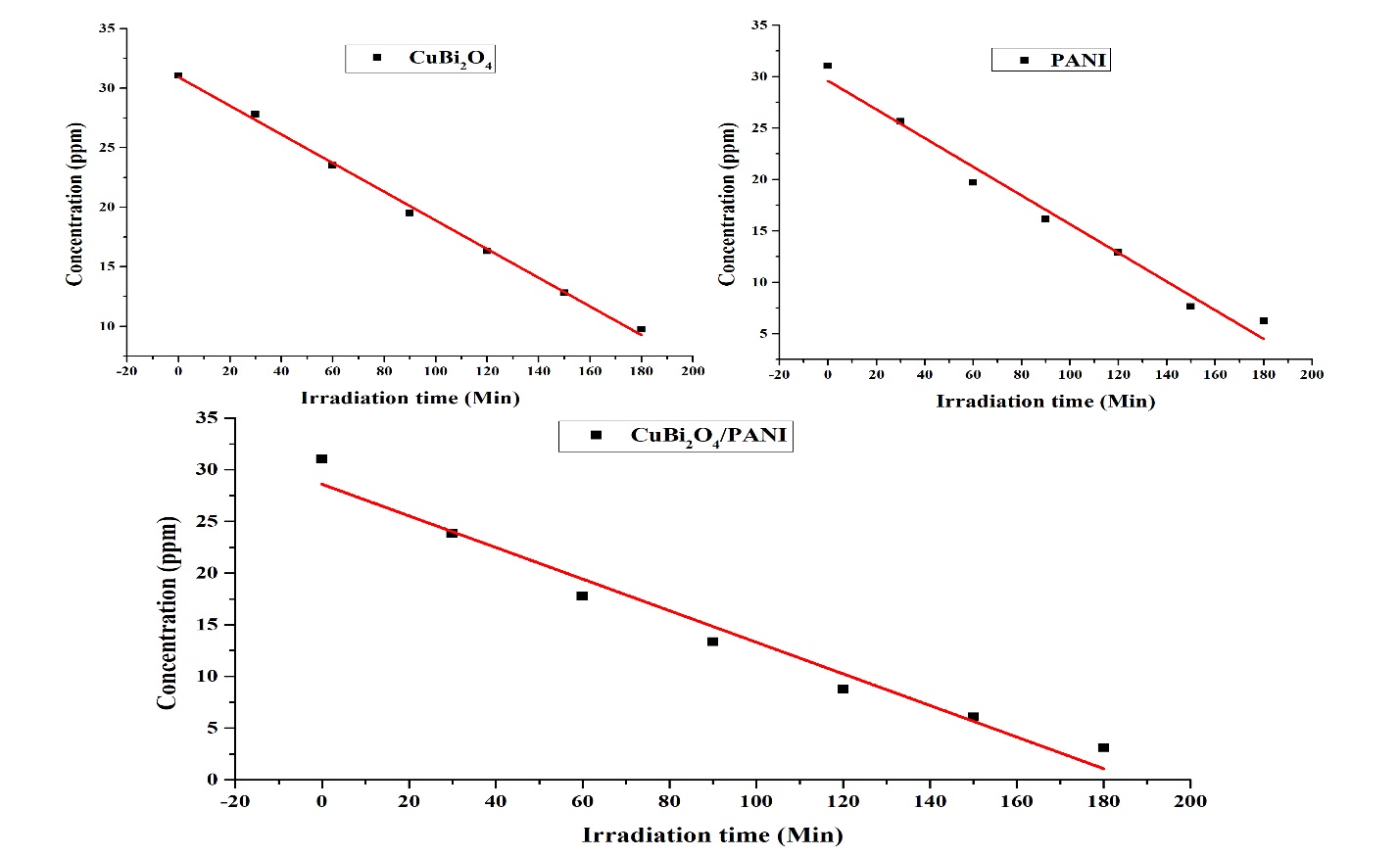


**Fig. S3**. Plots of C vs time shows the correlation of the data by regression analysis
